# Morphological Features of the Porcine Lacrimal Gland and Its Compatibility for Human Lacrimal Gland Xenografting

**DOI:** 10.1371/journal.pone.0074046

**Published:** 2013-09-12

**Authors:** Robert Henker, Michael Scholz, Simone Gaffling, Nagayoshi Asano, Ulrike Hampel, Fabian Garreis, Joachim Hornegger, Friedrich Paulsen

**Affiliations:** 1 Institute of Anatomy 2, University of Erlangen-Nuremberg, Erlangen, Germany; 2 Pattern Recognition Lab, University of Erlangen-Nuremberg, Erlangen, Germany; 3 Erlangen Graduate School in Advanced Optical Technologies (SAOT), University of Erlangen-Nuremberg, Erlangen, Germany; 4 Research and Development Center, Santen Pharmaceutical Co. Ltd, Nara, Japan; Bascom Palmer Eye Institute, University of Miami School of Medicine;, United States of America

## Abstract

In this study, we present first data concerning the anatomical structure, blood supply and location of the lacrimal gland of the pig. Our data indicate that the porcine lacrimal gland may serve as a potential xenograft candidate in humans or as an animal model for engineering of a bioartificial lacrimal gland tissue construct for clinical application. For this purpose, we used different macroscopic preparation techniques and digital reconstruction of the histological gland morphology to gain new insights and important information concerning the feasibility of a lacrimal gland transplantation from pig to humans in general. Our results show that the lacrimal gland of the pig reveals a lot of morphological similarities to the analogous human lacrimal gland and thus might be regarded as a xenograft in the future. This is true for a similar anatomical location within the orbit as well as for the feeding artery supply to the organ. Functional differences concerning the composition of the tear fluid, due to a different secretory unit distribution within the gland tissue will, however, be a challenge in future investigations.

## Introduction

Dry eye syndrome (DES), also known as keratoconjunctivitis sicca, is a common complaint with a high prevalence of 5.5% up to 33.7% depending on inclusion criteria and world region [1]. The DEWS report defined dry eye as a “multifactorial disease of the tears and ocular surface that results in symptoms of discomfort, visual disturbance, and tear film instability with potential damage to the ocular surface” [1]. Although DES may be caused by a deficiency in the aqueous component of tears, it is more commonly associated with hyperevaporation of tears – evaporative dry eye [1], [2], [3], [4]. Meibomian gland dysfunction (MGD), which refers to a diffuse abnormality of the meibomian glands, is considered to be the most common cause of evaporative dry eye [2], [3], but it may also be responsible for inflammatory conditions of the eyelids that do not necessarily manifest as classic DES [5]. Besides evaporative dry eye, aqueous tear deficiency due to lacrimal gland insufficiency is one of the major causes of dry eye. In severe cases such as Sjoegren’s syndrome, Stevens-Johnson syndrome or ocular cicatrical pemphigoid, therapy with artificial tears is often insufficient to relieve severe discomfort, prevent progressive ocular surface disease or enable visual rehabilitation by corneal transplantation.

The conventional and main approach to treatment of severe DES is to provide lubricating eye drops or tear substitutes. New treatment approaches are to target the underlying cause of DES instead of conventional symptomatic relief. One possibility of treating a deficiency in the aqueous component of tears can be to use a graft as a source of substitute lubrication. The idea of replacing an insufficient lacrimal gland is not new. Several ophthalmosurgeons accomplished a transplantation of the autologous submandibular gland into the temporal fossa. This procedure is capable of improving comfort, but the ocular surface remains damaged due to the salivary character of the new tear film [6], [7], [8], [9], [10], [11], [12], [13], [14], [15]. Also, more than 40% of patients show epiphora within 3–6 months after transplantation [16]. A graft for DES treatment has to meet several requirements. It should produce an adequate amount of tear fluid with a constant rate of secretion to avoid exsiccation or epiphora. The content of the fluid has to be similar not only in its enzymatic, but also in its electrolyte composition. To avoid pharmacological tear substitutes, the pre-ocular tear film has to be stable in the long term. The graft should also concur in morphological aspects such as size, excretory ducts and blood supply.

Here we present for the first time a morphological investigation of the lacrimal gland of the pig with a view to blood supply and lacrimal duct distribution using corrosion casts of the supplying arterial vessels and a 3D reconstruction of the excretory duct system. We intend to find similarities to the human lacrimal gland supply and thus a potential use of the lacrimal gland of the pig as a xenograft for transplantation in humans or as a model system to develop a bioartificial human lacrimal gland construct in the long term for clinical application.

## Materials and Methods

### Ethics Statement

For the morphological and histological examinations of porcine lacrimal glands, sagittally dissected heads of male and female domestic pigs (*Sus scrofa domestica*, all less than one year of age) were obtained from Unifleisch GmbH & Co KG (Erlangen, Germany). For the cast preparations we used 7 dissected porcine heads and for the macroscopic preparation 4 (5 male, 6 female). Unifleisch GmbH & Co KG (Erlangen, Germany) gave us unrestricted permission to use these animal parts for research. All experiments were performed in accordance with the ARVO Statement on Use of Animals in Ophthalmic and Vision Research.

### Corrosion Cast Preparation

A straight and buttoned irrigation cannula (UMI Germany; item number 315120) was inserted into the maxillary artery (MA) in order to free its branches of blood by flushing with NaCl solution. Once the nutritive MA branch of the lacrimal gland was blood-free, another cannula was inserted to allow permanent access for vascular casting. Technovit 7143 plastic compound (Heraeus Kulzer GmbH, Wehrheim, Germany) was prepared for injection by combining a partially polymerized monomer base with a catalyst (Technovit Universal Liquid, Heraeus Kulzer GmbH) at a ratio of 1∶1 [17]. A 20 ml syringe (MSG Wuppertal, Germany; item number 09203184) was used to inject the solution under hand pressure until the entire arterial system was filled [18]. The compound was allowed to polymerize for 24 hours. To remove the surrounding tissues, the heads were submerged in 30% HCl for 48 hours. The vascular casts obtained were rinsed in distilled water and finally photographed (Canon EOS 5D, Canon Inc., Tokyo, Japan).

### Macroscopic Examination

Sagittally dissected pig heads were fixed in a 4% formaldehyde solution in phosphate buffer for six months. For visualization of anatomical structures and a correlation between vessels supplying the lacrimal gland with blood and surrounding tissues, the heads were dissected macroscopically and relevant arteries were marked with red ink (Rohrer & Klinger OHG, Germany; item number 29700) using a brush.

### 3D Reconstruction of the Excretory Duct System

#### Histological preparation

Lacrimal glands (n = 2) were fixed in 4% formalin for 96 hours and afterwards embedded in paraffin. Tissue blocks were sectioned and applied on glass slides. 7 µm paraffin sections were deparaffinized and stained with hematoxylin and eosin. The sections were examined by bright field microscopy (BZ-9000E, Keyence Corporation, Osaka, Japan) and documented with a digital camera Canon EOS 450D. The images were captured (BZ-Analyzer, Keyence Corporation) and transferred to the Pattern Recognition Lab.

The preparation of histological sections requires extensive mechanical and chemical treatment of the respective tissue. The finally digitized histological images consequently show many artifacts. This includes, e.g., intensity inhomogeneities, low contrast, and non-linear tissue deformations due to the cutting procedure. All of these artifacts negatively impact the reconstruction result. Simply stacking up the images is therefore not an option. Therefore, several processing steps were performed which efficiently minimized these artifacts and restored the original lacrimal gland morphology.

#### Preprocessing

Due to unavoidable differences in slice thickness, temperature conditions and duration of chemical treatment during staining, the color intensities of the slices may differ. As a result, the same tissue types do not have the same intensity in different slice images. To reverse this effect, histogram matching was performed. A reference slice for the normalization of intensities is manually chosen and the associated image histogram is then used as the reference for linear matching to the histograms of the other slices, resulting in equal intensities for the same anatomical features.

#### Rigid registration

Cutting slices from the tissue block and subsequent attachment of the tissue slices to glass plates necessarily leads to differences in tissue orientation. To realign the entire slice sequence manually is a tedious and error-prone procedure in view of the high level of accuracy required. Therefore, an automated approach was chosen. Starting from an initial low-resolution version of two images, the rotation angle and translation are calculated for maximum image matchup. The parameters are then successively refined in higher-resolution versions of the images until the final best values are found. Although the slices are usually properly aligned after rigid registration, the connectivity of structures such as the lacrimal glands is in most cases not yet restored. The reason for this is that the slices are deformed during cutting, destroying the spatial coherence of the morphology. These deformations are usually reversed using non-rigid registration methods.

#### Reversal of non-linear deformations

In general, non-rigid registration methods calculate a translation vector for each image pixel for best fit of the transformed image to another image. Structures that belong together are moved such that their positions match again. Doing this for the whole image stack restores the connectivity of, e.g., vessel-like structures.

The deformation that is allowed for this purpose has to fulfill two main criteria. One is obviously that the images after the deformation must be more similar than before. A second mandatory criterion, however, is that the calculated deformation should emulate the real deformation of the tissue slices during cutting. It should therefore be smooth, without abrupt changes of direction.

As we are dealing with an entire sequence of images, this step has to be applied with great care. As explained in detail elsewhere [19], conventional non-rigid registration of histological image sequences tends to straighten curved structures - known as the “banana problem” [20]. To prevent this unwanted behavior, we used a specific scheme to apply non-rigid registration to the image sequence.

#### Stacking, visualization and segmentation

After processing of the entire histological image sequence, the individual images can be stacked together to compose the final volume image of the original tissue and loaded into a volume rendering tool for final visualization in 3-D. The spatial relations of the structures of interest can be further enhanced by segmenting the structures. We therefore semi-automatically segmented the tear ducts and the corresponding gland tissue using the free segmentation tool ITK-SNAP [21].

## Results

### Morphological Analysis

The porcine lacrimal gland lies in the superior-lateral angle of the orbit at its very margin. It is situated between the dorsal oblique muscle and the lateral rectus muscle. The average dimensions are 3.4 cm (dorsal-ventral)×2.2 cm (caudal-cranial)×1.2 cm (thickness). At dissection, the lacrimal gland appears as a soft and pale structure, which was macroscopically difficult to distinguish from the surrounding connective tissue.

### Arterial Blood Supply of the Porcine Lacrimal Gland

**Figure 1:**
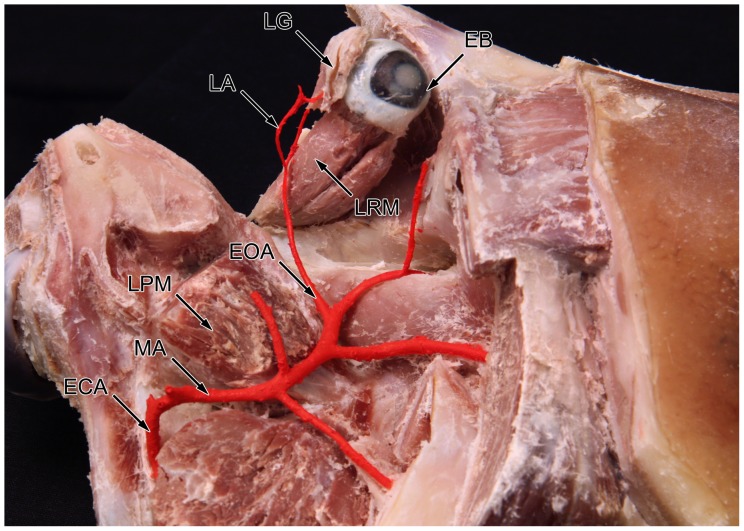
Sagittal dissected pig head fixed in formaldehyde solution. The artery blood supply of the lacrimal gland is shown as the lateral view on the right side of the head. ECA: external carotid artery; MA: maxillary artery; LPM: lateral pterygoid muscle; EOA: external ophthalmic artery; EB: eyeball; LRM: lateral rectus muscle; LA: lacrimal artery; LG: lacrimal gland.

**Figure 2:**
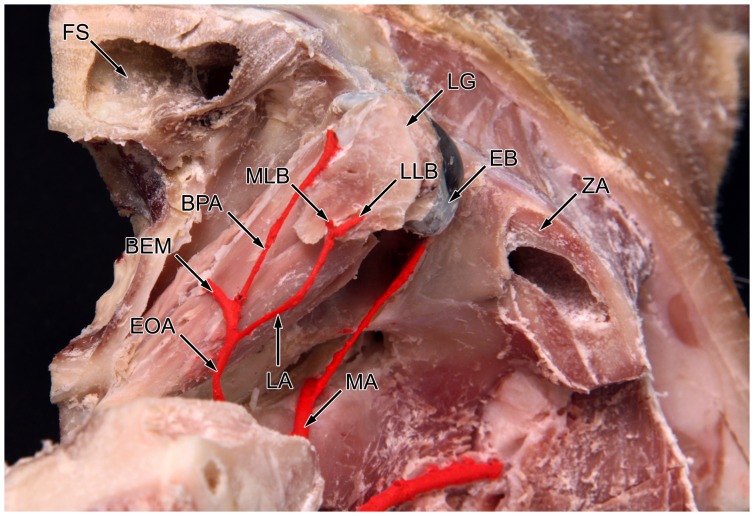
Sagittal dissected pig head fixed in formaldehyde solution. The artery blood supply of the lacrimal gland in the dorsal view on the right side of the head. MA: maxillary artery; EOA: external ophthalmic artery; EB: eyeball; LA: lacrimal artery; LG: lacrimal gland; MLB: medial lacrimal branch; LLB: lateral lacrimal branch; BEM: branch for extraocular muscles; BPA: branch for palpebral area; FS: frontal sinus; ZA: zygomatic arch.

**Figure 3:**
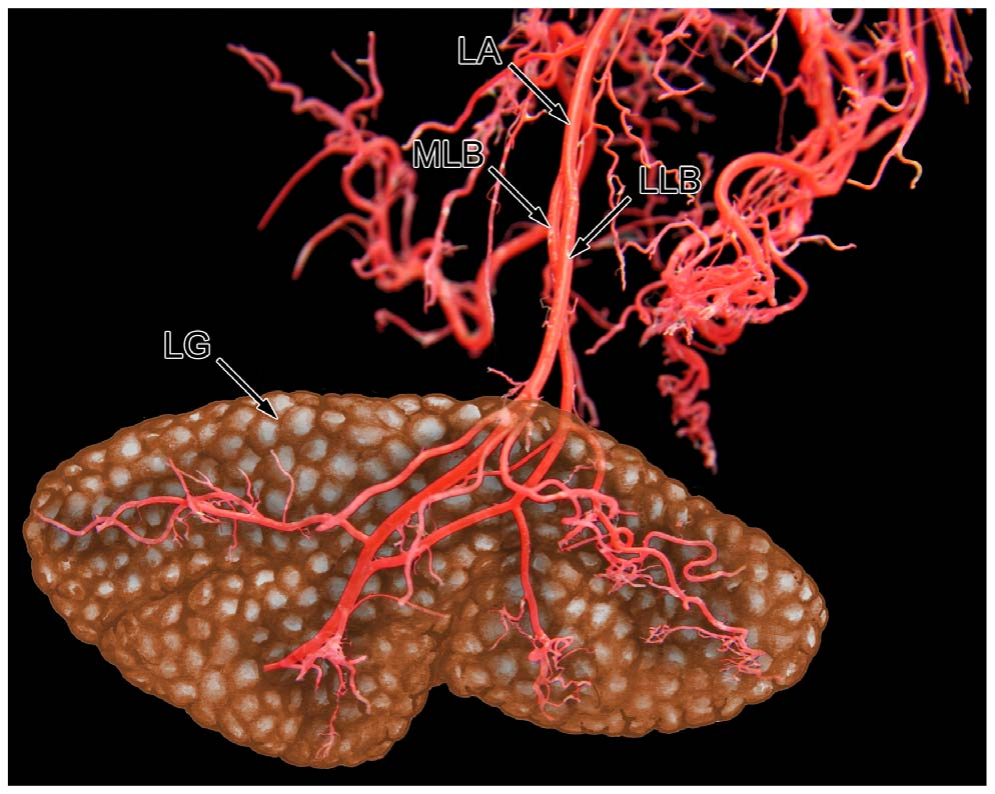
Vascular corrosion cast of the lacrimal artery with intraglandular artery divisions. The lacrimal gland was added as a graphic illustration to get a steric impression of the blood supply. Frontal view on the left lacrimal artery. LA: lacrimal artery; MLB: medial lacrimal branch; LLB: lateral lacrimal branch; LG: lacrimal gland.

The arterial blood supply of the lacrimal gland originates from the external carotid artery that continues in the maxillary artery. At the level of the orbita, a branch diverges from the maxillary artery running along the rostral side of the lateral pterygoid muscle (Fig. 1). This branch is described as a. ophthalmica externa [22]. After making a sharp bend in the posterior part of the orbit it splits into three different branches supplying the extraocular muscles, the palpebral area and the lacrimal gland (Fig. 2). The lacrimal artery runs along the upper margin of the lateral rectus muscle and divides into two branches before entering the gland. The medial lacrimal branch is responsible for the vascular supply of the deep and lower parts of the gland. The lateral lacrimal branch ensures nutrition of the superficial and upper parts of the gland (Fig. 3).

### Excretory Duct System

**Figure 4:**
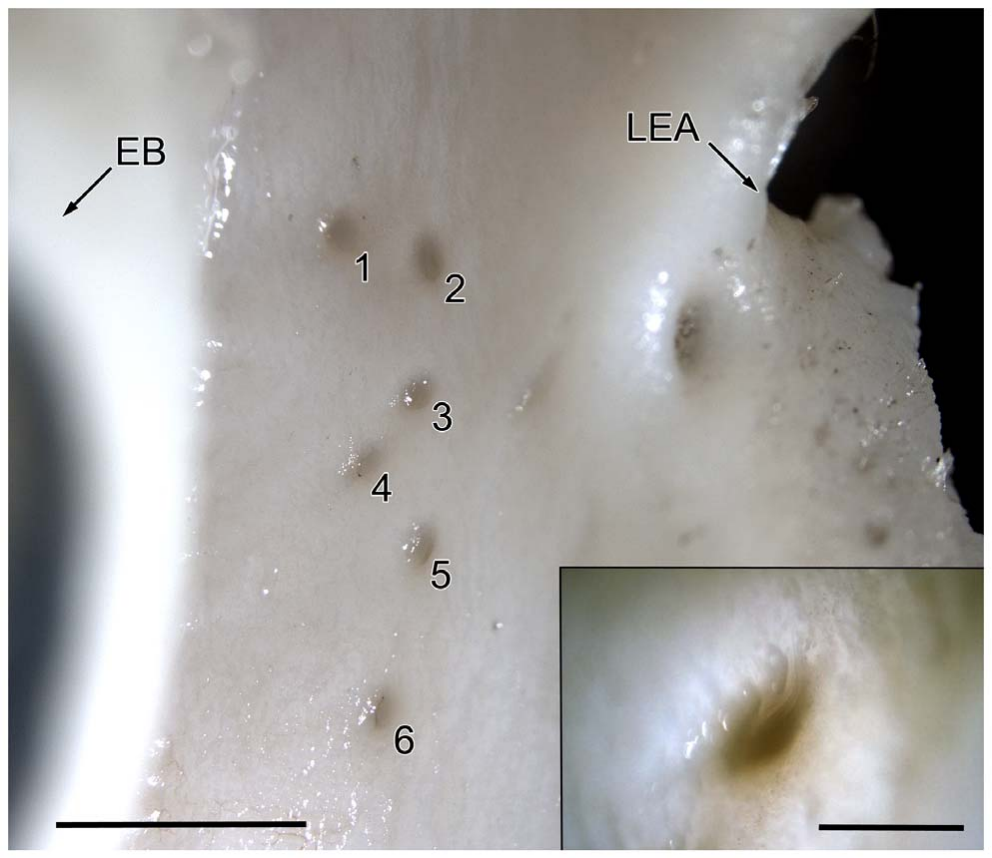
Excretory ducts of the left lacrimal system in pig. Conjunctival view at the level of the eye angle, left scale bar: 2 mm. EB: eyeball; LEA: left eye angle; 1–6: excretory ducts. Right bottom square: higher magnification of an excretory duct, scale bar: 200 µm.

There are about seven main excretory ducts responsible for transporting lacrimal fluids to the ocular surface. They end in the fornix conjunctivae at the level of the lateral eye angle. The ducts show a mean diameter of 200 µm (Fig. 4).

### 3-D Reconstruction of Histological Sections

**Figure 5:**
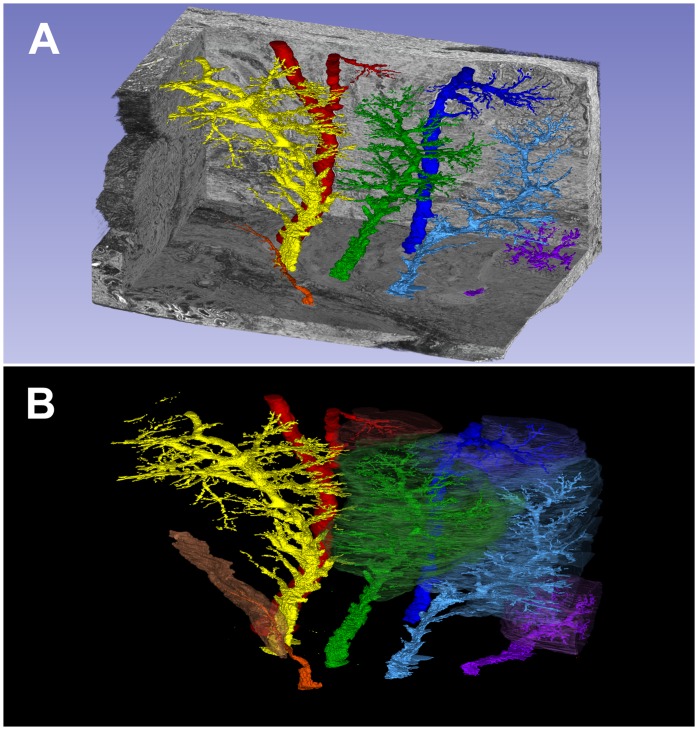
3D reconstruction by digitized and computer based processed histological sections of the pig lacrimal gland. A) Visualization of seven excretory lacrimal ducts (different colors) within the paraffin embedded lacrimal gland (grey). B) Separate exposure of each lacrimal duct in different colors with surrounding gland tissue reconstruction. Only the yellow-marked duct system is displayed alone, without gland tissue reconstruction.

The reconstructed volume shows seven excretory ducts, which were semi-automatically segmented using ITK-SNAP [21] (Fig. 5). The number and morphology of the excretory lacrimal duct system in pigs corresponds closely to the analogous human organ, in which six to twelve excretory lacrimal ducts are generally found.

## Discussion

Pigs can be regarded today as the most promising option for organ xenotransplantation. As a potential donor species they feature qualities similar in physiology and convenient availability [23], [24]. In some areas of medicine (islet cell transplants, cornea transplants, heart valve replacement, blood transfusions), we already have knowledge of and experience with foreign tissue transplantation [25], [26], [27], [28]. However, serious immunological barriers still prevent whole organ pig-to-human transplantation. The required immunosuppression will lead to significantly higher complication rates in terms of infections and malignancies in patients [29]. The breeding of so-called “genetically engineered pigs” should prevent hyperacute tissue rejection in the future [30], [31], [32]. The prevention of subsequent tissue rejection after xenotransplantation (acute vascular, acute humoral and chronic rejection) thus remains an urgent goal of research. Another risk already observed is the possible transfer of endogenous pig retroviruses (PERVs - porcine endogenous retrovirus) to human cells. However, this is currently regarded as a problem of minor significance [33], [34], [35].

### Arterial Blood Supply

In most cases the human lacrimal gland receives its vascular supply from a collateral branch of the ophthalmic artery, the lacrimal artery.

Hayreh et al. classified the origin and course of the human lacrimal artery into three groups. In the type I variety, the most frequent, the lacrimal artery is a branch of the ophthalmic artery. In this case, the lacrimal artery originates from the ophthalmic artery and runs along the margin of the rectus lateralis muscle. In type II, the lacrimal artery originates from the middle meningeal artery. In type III the gland is simultaneously supplied by two arteries, one originating from the ophthalmic artery and the other from the middle meningeal artery. In this case, the lacrimal gland is the site of an intraorbital anastomosis between the internal and external carotid systems [36].

Erdogmus et al. also reported on the human lacrimal artery and its glandular branches. The lacrimal artery ran laterally and then bent forward over the upper margin of the lateral rectus muscle to reach the lacrimal gland. The lacrimal artery can be very tortuous. Variability of the glandular branch in its course toward the lacrimal gland were distinguished in three types. In type I, which is the most frequent, the glandular branch is a single trunk supplying the lacrimal gland. The lacrimal artery penetrated centrally into the lacrimal gland and divided into two or four branches. One of them ran centrally, and the remaining branches ran along the borders of the gland to its poles. In type II, the glandular branch ran as two branches, immediately after leaving the lacrimal artery. In type III, the glandular branch ran as three branches immediately after leaving the lacrimal artery, dividing into small branches upon entering the gland [37].

In all our specimens, the lacrimal artery originated from the external ophthalmic artery and a single glandular branch divides in two small branches before entering the gland. Excepting anatomical variations, we can state that both human and porcine anatomy show a single-trunk origin of the lacrimal artery. A vascular connection should be feasible for xenotransplantation. The intraglandular arterial variability depends on the type of artery division.

### Morphological Analysis

According to Kleckowska–Nawrot, the porcine lacrimal gland is situated inside the periorbit in the dorsolateral angle of the eye [38]. According to Getty, the lacrimal gland in the adult pig is triangular in shape, but it is not as large as in other domestic species [39]. Tamboli et al. reported on the normal range of Caucasian lacrimal gland dimensions seen on CT. Mean lacrimal gland axial length in right orbits was 14.7 mm and 14.5 mm in left orbits. Coronal length averaged 17.7 mm in right eyes and 16.9 mm in left eyes [40]. In comparison, the lacrimal gland in the adult pig seems to be larger by 50% (width) to 90% (length).

The examinations of the lacrimal gland ultrastructure in the adult pig conducted by Kühnel and Scheele showed that alveoli and tubules are composed of serous–mucous cells, serous cells and mucous cells, with a predominance of serous-mucous cells. The excretory ducts are composed of a simple cuboid epithelium, under which myoepithelial cells are situated [41].

In summary, we can state that the lacrimal gland of the pig generally shows a high level of morphological similarity to the human lacrimal gland regarding the aspects examined in this study. As to xenotransplantation, the complete transposition of the lacrimal gland from pigs to humans seems to be conceivable, due to similar anatomical location and relationship to the bony skull and conjunctiva. The connection to the human vascular system would also be quite uncomplicated, since in both species the lacrimal gland has only one feeding artery supplying the organ. The secretory capacity of the lacrimal gland in pigs can only be assumed on the basis of histomorphological images. Histological and, thus functional differences, are expected due to the large number of seromucous secretory units in pig, whereas in humans mainly serous-secreting acini appear in the lacrimal gland tissue. At present, however, it has to be stated that the xenografting options concerning the transplantation of the lacrimal gland from pig to human remain speculative. This is true because the current state of research does not provide sufficient information on distribution and composition of immune cell populations and the immune system of the porcine lacrimal gland itself, respectively [42]. To meet these challenges, the exudate composition analysis of porcine lacrimal fluid and comparison to the human equivalent as well as detailed analysis of the venous blood and lymphatic drainage system of the porcine lacrimal gland are focal points for future investigations.

## References

[1] WorkShop SotIDE (2007) Research in dry eye: report of the Research Subcommittee of the International Dry Eye WorkShop. Ocul Surf 5: 179–193. 17508121 10.1016/s1542-0124(12)70086-1

[2] Tong L, Chaurasia SS, Mehta JS, Beuerman RW (2010) Screening for meibomian gland disease: its relation to dry eye subtypes and symptoms in a tertiary referral clinic in singapore. Invest Ophthalmol Vis Sci 51: 3449–3454. 20181848 10.1167/iovs.09-4445

[3] Schaumberg DA, Nichols JJ, Papas EB, Tong L, Uchino M, et al (2011) The international workshop on meibomian gland dysfunction: report of the subcommittee on the epidemiology of, and associated risk factors for, MGD. Invest Ophthalmol Vis Sci 52: 1994–2005. 21450917 10.1167/iovs.10-6997e PMC3072161

[4] King-Smith PE, Nichols JJ, Braun RJ, Nichols KK (2011) High resolution microscopy of the lipid layer of the tear film. Ocul Surf 9: 197–211. 22023815 10.1016/s1542-0124(11)70033-7 PMC4313928

[5] Blackie CA, Korb DR, Knop E, Bedi R, Knop N, et al (2010) Nonobvious obstructive meibomian gland dysfunction. Cornea 29: 1333–1345. 20847669 10.1097/ICO.0b013e3181d4f366

[6] Murube-del-Castillo J (1986) Transplantation of salivary gland to the lacrimal basin. Scand J Rheumatol Suppl 61: 264–267. 3473641

[7] Kumar PA, Macleod AM, O’Brien BM, Hickey MJ, Knight KR (1990) Microvascular submandibular gland transfer for the management of xerophthalmia; an experimental study. Br J Plast Surg 43: 431–436. 1697488

[8] MacLeod AM, Robbins SP (1992) Submandibular gland transfer in the correction of dry eye. Aust N Z J Ophthalmol 20: 99–103. 1389142 10.1111/j.1442-9071.1992.tb00719.x

[9] Geerling G, Sieg P, Meyer C, Bastian GO, Laqua H (1998) Transplantation of autologous submandibular glands in very severe keratoconjunctivitis sicca. 2 year outcome. Ophthalmologe 95: 257–265. 9623264 10.1007/s003470050272

[10] Geerling G, Sieg P, Bastian GO, Laqua H (1998) Transplantation of the autologous submandibular gland for most severe cases of keratoconjunctivitis sicca. Ophthalmology 105: 327–335. 9479295 10.1016/s0161-6420(98)93406-6

[11] Geerling G, Honnicke K, Schroder C, Framme C, Sieg P, et al (1999) Quality of salivary tears following autologous submandibular gland transplantation for severe dry eye. Graefes Arch Clin Exp Ophthalmol 237: 546–553. 10424304 10.1007/s004170050277

[12] Geerling G, Honnicke K, Schroder C, Framme C, Sieg P, et al (2000) Quality of salivary tears following autologous submandibular gland transplantation for severe dry eye. Graefes Arch Clin Exp Ophthalmol 238: 45–52. 10664052 10.1007/s004170050008

[13] Geerling G, Sieg P (2008) Transplantation of the major salivary glands. Dev Ophthalmol 41: 255–268. 18453774 10.1159/000131094

[14] Jacobsen HC, Hakim SG, Lauer I, Dendorfer A, Wedel T, et al (2008) Long-term results of autologous submandibular gland transfer for the surgical treatment of severe keratoconjunctivitis sicca. J Craniomaxillofac Surg 36: 227–233. 18406158 10.1016/j.jcms.2007.08.010

[15] Ge XY, Yu GY, Fu J, Wu DC, Zhang XX, et al (2012) An experimental study of the management of severe keratoconjunctivitis sicca with autologous reduced-sized submandibular gland transplantation. Br J Oral Maxillofac Surg 50: 562–566. 22051179 10.1016/j.bjoms.2011.10.004

[16] Ding C, Zhang Y, Peng X, Wang Y, Zhang L, et al (2011) Proteomic analysis of human transplanted submandibular gland in patients with epiphora after transplantation. J Proteome Res 10: 2206–2215. 21384922 10.1021/pr100965q

[17] Cordes J (1989) Korrosionstechnik. Der Präparator 35: 21–28.

[18] Lehmann KS, Ritz JP, Valdeig S, Schenk A, Holmer C, et al (2008) Portal vein segmentation of a 3D-planning system for liver surgery–in vivo evaluation in a porcine model. Annals of surgical oncology 15: 1899–1907. 18449610 10.1245/s10434-008-9934-x

[19] Malandain G, Bardinet E, Nelissen K, Vanduffel W (2004) Fusion of autoradiographs with an MR volume using 2-D and 3-D linear transformations. NeuroImage 23: 111–127. 15325358 10.1016/j.neuroimage.2004.04.038

[20] Streicher J, Weninger WJ, Muller GB (1997) External marker-based automatic congruencing: a new method of 3D reconstruction from serial sections. The Anatomical record 248: 583–602. 9268147 10.1002/(SICI)1097-0185(199708)248:4<583::AID-AR10>3.0.CO;2-L

[21] Yushkevich PA, Piven J, Hazlett HC, Smith RG, Ho S, et al (2006) User-guided 3D active contour segmentation of anatomical structures: significantly improved efficiency and reliability. NeuroImage 31: 1116–1128. 16545965 10.1016/j.neuroimage.2006.01.015

[22] Popesko P (1989) Atlas der topographischen Anatomie der Haustiere 2.: Brust- und Bauchhöhle: Enke.

[23] Kobayashi E, Hishikawa S, Teratani T, Lefor AT (2012) The pig as a model for translational research: overview of porcine animal models at Jichi Medical University. Transplantation research 1: 8. 23369409 10.1186/2047-1440-1-8 PMC3560993

[24] Tanaka H, Kobayashi E (2006) Education and research using experimental pigs in a medical school. Journal of artificial organs : the official journal of the Japanese Society for Artificial Organs 9: 136–143. 16998697 10.1007/s10047-006-0343-2

[25] van der Windt DJ, Bottino R, Casu A, Campanile N, Smetanka C, et al (2009) Long-term controlled normoglycemia in diabetic non-human primates after transplantation with hCD46 transgenic porcine islets. American journal of transplantation : official journal of the American Society of Transplantation and the American Society of Transplant Surgeons 9: 2716–2726. 10.1111/j.1600-6143.2009.02850.x 19845582

[26] Kim MK, Wee WR, Park CG, Kim SJ (2011) Xenocorneal transplantation. Current opinion in organ transplantation 16: 231–236. 21415826 10.1097/MOT.0b013e328344870c

[27] Manji RA, Menkis AH, Ekser B, Cooper DK (2012) Porcine bioprosthetic heart valves: The next generation. American heart journal 164: 177–185. 22877802 10.1016/j.ahj.2012.05.011

[28] Long C, Hara H, Pawlikowski Z, Koike N, d’Arville T, et al (2009) Genetically engineered pig red blood cells for clinical transfusion: initial in vitro studies. Transfusion 49: 2418–2429. 19624491 10.1111/j.1537-2995.2009.02306.x

[29] Ekser B, Cooper DK (2010) Overcoming the barriers to xenotransplantation: prospects for the future. Expert review of clinical immunology 6: 219–230. 20402385 10.1586/eci.09.81 PMC2857338

[30] Cozzi E, White DJ (1995) The generation of transgenic pigs as potential organ donors for humans. Nature medicine 1: 964–966. 10.1038/nm0995-964 7585226

[31] Cooper DK, Koren E, Oriol R (1993) Genetically engineered pigs. Lancet 342: 682–683. 8103167 10.1016/0140-6736(93)91791-j

[32] Phelps CJ, Koike C, Vaught TD, Boone J, Wells KD, et al (2003) Production of alpha 1,3-galactosyltransferase-deficient pigs. Science 299: 411–414. 12493821 10.1126/science.1078942 PMC3154759

[33] Patience C, Patton GS, Takeuchi Y, Weiss RA, McClure MO, et al (1998) No evidence of pig DNA or retroviral infection in patients with short-term extracorporeal connection to pig kidneys. Lancet 352: 699–701. 9728987 10.1016/S0140-6736(98)04369-4

[34] Paradis K, Langford G, Long Z, Heneine W, Sandstrom P, et al (1999) Search for cross-species transmission of porcine endogenous retrovirus in patients treated with living pig tissue. The XEN 111 Study Group. Science 285: 1236–1241. 10455044 10.1126/science.285.5431.1236

[35] Fishman JA, Scobie L, Takeuchi Y (2012) Xenotransplantation-associated infectious risk: a WHO consultation. Xenotransplantation 19: 72–81. 22497509 10.1111/j.1399-3089.2012.00693.x PMC3768267

[36] Hayreh SS, Dass R (1962) The Ophthalmic Artery: Ii. Intra-Orbital Course. Br J Ophthalmol 46: 165–185. 18170768 10.1136/bjo.46.3.165 PMC510181

[37] Erdogmus S, Govsa F (2005) Importance of the anatomic features of the lacrimal artery for orbital approaches. J Craniofac Surg 16: 957–964. 16327539 10.1097/01.scs.0000179741.68294.1c

[38] Kleckowska-Nawrot J, Dziegiel P (2008) Morphology of lacrimal gland in pig fetuses. Anat Histol Embryol 37: 74–77. 18197904 10.1111/j.1439-0264.2007.00798.x

[39] Getty R (1975) Sisson and Grossmańs The Anatomy of the Domestic Animals. Philadelphia, London, Toronto: W. B. Saunders Company.

[40] Tamboli DA, Harris MA, Hogg JP, Realini T, Sivak-Callcott JA (2011) Computed tomography dimensions of the lacrimal gland in normal Caucasian orbits. Ophthal Plast Reconstr Surg 27: 453–456. 10.1097/IOP.0b013e31821e9f5d 21659915

[41] Kühnel WS, G Scheele (1979) Zur Feinstruktur der glandula lacrimalis des Schweins Anat Anz. 145: 87–106. 434482

[42] Aitken ID, Survashe BD (1977) Plasma cells in vertebrate paraocular glands. International archives of allergy and applied immunology 53: 62–67. 838515 10.1159/000231732

